# A Dual Electrode Biosensor for Glucose and Lactate Measurement in Normal and Prolonged Obese Mice Using Single Drop of Whole Blood

**DOI:** 10.3390/bios11120507

**Published:** 2021-12-09

**Authors:** Mukesh Thapa, Ryong Sung, Yun Seok Heo

**Affiliations:** Department of Biomedical Engineering, School of Medicine, Keimyung University, 1095 Dalgubeol-daero, Dalseo-gu, Daegu 42601, Korea; 1004672@stu.kmu.ac.kr (M.T.); rs.ryongs@gmail.com (R.S.)

**Keywords:** obesity, electrochemical biosensor, dual electrodes, glucose, lactate

## Abstract

Understanding the levels of glucose (G) and lactate (L) in blood can help us regulate various chronic health conditions such as obesity. In this paper, we introduced an enzyme-based electrochemical biosensor adopting glucose oxidase and lactate oxidase on two working screen-printed carbon electrodes (SPCEs) to sequentially determine glucose and lactate concentrations in a single drop (~30 µL) of whole blood. We developed a diet-induced obesity (DIO) mouse model for 28 weeks and monitored the changes in blood glucose and lactate levels. A linear calibration curve for glucose and lactate concentrations in ranges from 0.5 to 35 mM and 0.5 to 25 mM was obtained with R-values of 0.99 and 0.97, respectively. A drastic increase in blood glucose and a small but significant increase in blood lactate were seen only in prolonged obese cases. The ratio of lactate concentration to glucose concentration (L/G) was calculated as the mouse’s gained weight. The results demonstrated that an L/G value of 0.59 could be used as a criterion to differentiate between normal and obesity conditions. With L/G and weight gain, we constructed a diagnostic plot that could categorize normal and obese health conditions into four different zones. The proposed dual electrode biosensor for glucose and lactate in mouse whole blood showed good stability, selectivity, sensitivity, and efficiency. Thus, we believe that this dual electrode biosensor and the diagnostic plot could be used as a sensitive analytical tool for diagnosing glucose and lactate biomarkers in clinics and for monitoring obesity.

## 1. Introduction

Glucose and lactate are vital bio-compounds in our metabolic activities. The measurement of glucose and lactate can help us figure out various disease prognoses and developments to ultimately manage personal health [1,2,3,4]. For instance, glucose is considered a key metabolic substrate for tissue energy production. Its concentration in blood has been traditionally used as a biomarker of diabetes, hypertension, and fatty liver [3,5,6,7]. Compared with blood glucose, blood lactate has attracted less attention. Blood lactate can still assist in the diagnosis of disease conditions such as pyruvate metabolism defects, sepsis, hypoxia, and meningitis [4,8]. In addition, monitoring the lactate level in the body has been recently used to manage the fatigue and endurance of athletes [5,6,8]. Although both glucose and lactate are major contributing factors to metabolic cycles, to date, they have been measured in different contexts of diseases. Few researchers have studied the combining effect of glucose and lactate [9]. Only in a small number of cases with prolonged and genetic medical history such as kidney and liver malfunction, insulin resistance, and thyroid have both glucose and lactate been reported to be elevated considerably [8,10,11]. Although glucose has a higher preference than lactate, some studies have suggested that lactate is an independent factor that should be given equal importance as glucose for monitoring obesity and type 2 diabetes [11].

**Figure 1:**
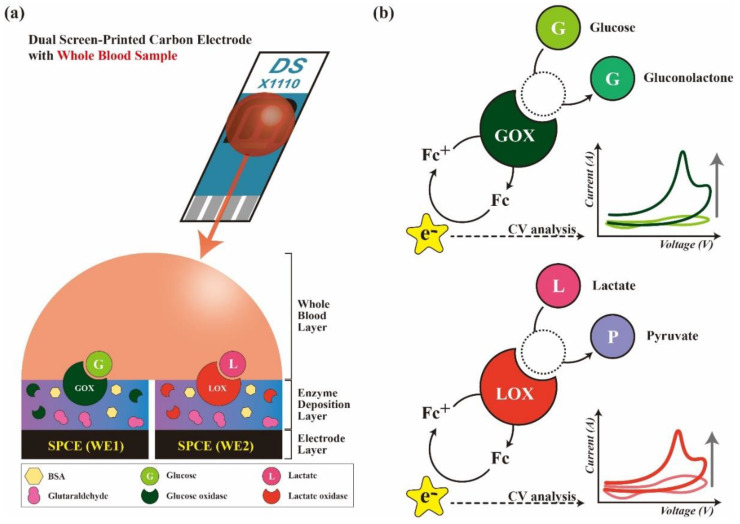
Schematic illustrations of (**a**) sample load on the DWE biosensor. All electrodes of DSPCEs (GOX) is deposited on WE1, and LOX is deposited on WE2) are covered with a single drop of whole blood (30 µL), and (**b**) mechanism of measuring electron (e^−^) productions from blood metabolites (glucose and lactate) on WE1 and WE2.

To develop a device for measuring glucose and lactate together, several attempts have been made. However, a user-friendly point-of-care device for measuring both glucose and lactate in whole blood is still lacking [12,13,14,15,16]. Conventionally, glucose and lactate are measured one by one (dual drops for sample loading) in different time frames (e.g., a time delay exists between drops compared with a single drop) using separate measurement protocols for glucose and lactate (e.g., electrochemical detection for glucose and spectrophotometry for lactate), which facilitates different sample status (e.g., whole blood for glucose vs. plasma for lactate). We need to enhance the shortcomings of these conventional methodologies to develop a single integrated device for measuring both glucose and lactate in a single drop of blood. Here, we introduce a double electrode biosensor (DEB) that can consecutively measure glucose and lactate in the same sample (single drop of whole blood with minimal time delay) on double working screen-printed carbon electrode (DWSPCE) using an electrochemical detection system. The working principle and electrochemical detection mechanism of our device are shown in Figure 1a,b.

Dual working electrode (DWE) was constructed using a simple drop cast deposition of glucose oxidase and lactate oxidase with mediators. To assess the efficacy of the proposed biosensor, we determined its stability, selectivity, sensitivity, and efficiency. Linear calibration plots were obtained within clinical ranges of 0.5–35 mM for glucose and 0.5–15 mM for lactate. Furthermore, the biosensor was evaluated with blood samples from normal and obese mice. We developed an obese mouse model fed by a high-fat diet (HFD) chow for the initial 12 weeks and then continued the diet (16 weeks) to obtain prolonged obese cases. With this obese model, both glucose and lactate showed measurable increases compared with in normal cases. For better and simple analysis, we suggested a new parameter, the ratio of lactate to glucose (L/G), to reflect the influence of both glucose and lactate as a single parameter. We also constructed a “diagnosis plot” for obesity using L/G associated with respective weight gain, which categorized mouse conditions onto four different zones and represented a meaningful difference between normal mice and obese mice. To the best of our knowledges, this is the first study to measure both glucose and lactate electrochemically in a single drop of whole blood and to construct a “diagnostic plot” for obesity monitoring.

## 2. Materials and Methods

### 2.1. Enzyme Deposition on Electrodes

Bovine serine albumin (BSA, 25 mg/mL) and glutaraldehyde (2.5% *w*/*v*) were mixed and then dissolved in distilled water at a 10:1 volume ratio. A mediator (FcMeOH) was added to the fore solution to obtain a final concentration of 0.3 mM. Either glucose oxidase (GOX) or lactate oxidase (LOX) was separately added to each mixture at a 5:1 volume ratio, and the resulting preparation was applied to cover the working electrodes [17]. The enzyme deposition method was initially implemented individually using either GOX or LOX along with single working screen-printed carbon electrodes. The method was validated through separate measurements for glucose and lactate, as shown in Supplementary Figure S1a,b. After validation, the methodology was applied for(DWE). Enzymes used to prepare the deposition solutions for DWE were optimized (500 U/mL for GOx and 300 U/mL for LOX), as shown in Supplementary Figure S2a,b. These enzyme solutions were deposited onto the first (WE1) and the second (WE2) working electrodes (WE1 for GOX and WE2 for LOX) and then dried at room temperature, to be used for all experiments.

### 2.2. Stability

The stability of the electrode was evaluated under the following seven conditions: (i) CV response of PBS on bare electrode, (ii) CV response of PBS on GOX-modified electrode, (iii) CV response of PBS on LOX-modified electrode, (iv) CV response of 20 mM glucose on bare electrode, (v) CV response of 20 mM glucose on GOX-modified electrode, (vi) CV response of 15 mM lactate on bare electrode, and (vii) CV response of 15 mM lactate on LOX-modified electrode. The operating conditions for CV measurements were a scan rate of 500 mV/s, sensitivity at 10 mA/V, quiet time of 2 s, a sample interval of 1 mV, an initial voltage of −200 mV, and a final voltage of 400 mV. The peak current (*I_p_*) was recorded as a representative value of the CV in each condition. Different scan rates (75, 125, 250, and 500 mV/s) were used to further characterize the effects of electron diffusion on stability.

Additional experiments were performed to evaluate the stability of the DWE performance under harsh conditions. The stability of the electrode was evaluated at different temperature storage conditions (for 24 h). DWE performances were also evaluated at varying pH levels. The storage temperature was maintained in either refrigerators or normal incubators. Neutral pH solution was prepared using phosphate buffer, whereas acidic and basic solutions were prepared using HCl and NaOH, respectively. The storage ability of DWE was also exmained for up to 9 days at 4 °C. Readings were collected on days 0, 3 (72 h), 6 (114 h), and 9 (216 h). All stability experiments were conducted using 15 mM lactate and 20 mM glusose standard solutions.

### 2.3. Selectivity

In total, eight combinations of glucose and lactate mixtures were evaluated to understand mutual interference. WE1 and WE2 were monitored to determine the selectivity of glucose and lactate, respectively. A standard solution of 20 mM glucose was individually combined with 0, 5, 10, and 15 mM lactate. A standard solution of 15 mM lactate was individually combined with 0, 10, 20, and 30 mM glucose. Mutual interference was examined by comparing the CV (*I_p_*) values of these eight solutions.

Different blood biometabolites including cholesterol (8 mM), uric acid (200 µM), pyruvate (1.5 mM), ascorbic acid (1 mM), and xylose (1 mM) were individually mixed with glucose (7 mM) and lactate (4 mM) in separate mixture. Subsequently, we investigated the ability of DWE in discriminating between glucose and lactate, in a mixture of bodily fluids by comparing the mean (*I_p_*) of the mixture with those of pure glucose and lactate solutions.

### 2.4. Sensitivity

Glucose and lactate calibration curves were independently obtained using a gradient of seven different concentrations: 0, 0.5, 2, 6, 10, 20, and 35 mM for glucose and 0, 0.5, 3, 6, 10, 15 and 25 mM for lactate. These seven glucose and lactate combinations (I–VII) were prepared as follows: I (0 mM glucose + 25 mM lactate), II (0.5 mM glucose + 15 mM lactate), III (2 mM glucose + 10 mM lactate), IV (6 mM glucose + 6 mM lactate), V (10 mM glucose + 3 mM lactate), VI (20 mM glucose + 0.5 mM lactate), and VII (35 mM glucose + 0 mM lactate). From I to VII, the glucose concentration was increased from its minimum (0 mM) to its maximum (35 mM), while the lactate concentration was decreased from its maximum (25 mM) to its minimum (0 mM). For each concentration, with the measurements were repeated three times. Consistency was ensured by performing glucose measurement first, followed by lactate measurement using cyclic voltammetry. The time interval between the glucose and lactate measurements was maintained at 20 s. Calibration curves were constructed for glucose and lactate. Using the slopes of these curves, the measurement sensitivity of DWE for glucose and lactate measurement was calculated.

### 2.5. Obesity Mice Model

An obesity model was established through diet-induced obesity (DIO) by feeding mice a diet with high-fat content [18]. The physiological changes in DIO mice were similar to those observed for obesity development in humans. DIO mice were selected for the obesity model because they are not genetically engineered [18]. In total, 14 C57BL/6J male mice were used for the experiment. Of these, 7 mice were fed a high fat diet (HFD) (5.4 kcal/g) and the other 7 were fed a normal diet (ND) (3.83 kcal/g) for 12 weeks; a gain in body weight was considered the establishment of obesity [18]. The obesity condition was prolonged by continuing the diet for 16 more weeks for the DIO group. The body weights of both normal and obese mice were measured after 28 weeks of initiating the diet. All animal experiments were approved and performed in accordance with the guidelines and regulations provided by the Institutional Animal Care and Use Committee at School of Medicine, Keimyung University (Mice IRB No: KM2019-18R2).

### 2.6. Glucose and Lactate Measurements Using Mouse Whole Blood

More than 150 µL of blood was collected from the submandibular vein of each mouse. A single drop (30 µL) of blood was used to cover all the electrodes, which included two working electrodes of the DWE biosensor. The concentrations of glucose and lactate were determined and used to construct a diagnostic plot of obesity based on lactate to glucose ratio.

All electrochemical measurements were performed using a CHI760E electrochemical workstation from CH Instruments (Austin, TX, USA) and a DWSPCE were purchased from Drop Sens.

## 3. Results and Discussion

### 3.1. Stability

**Figure 2:**
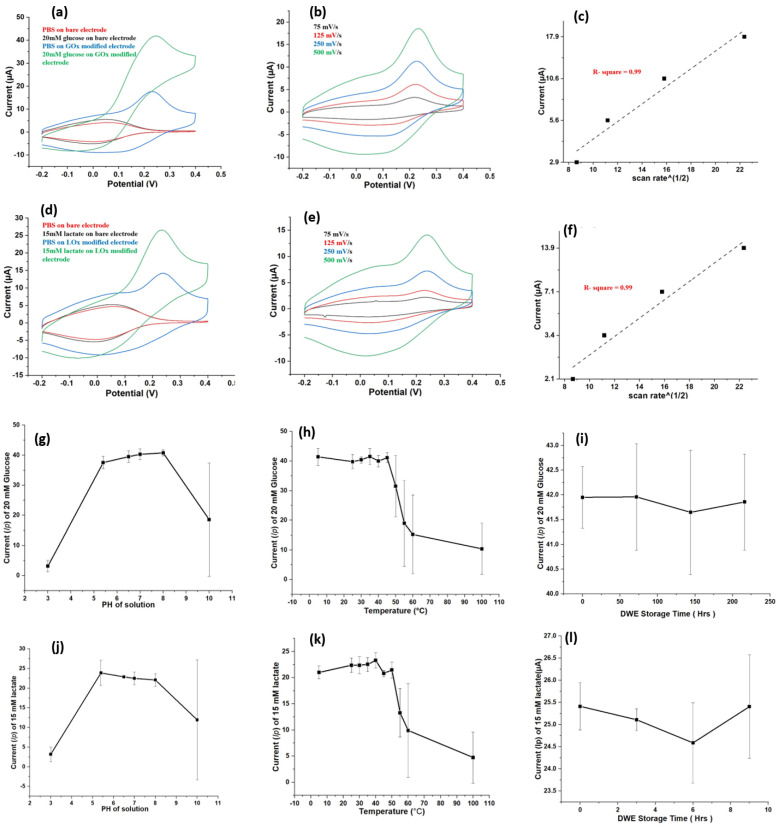
Stability test: (**a**) collective Cyclic voltammetry (CV) responses of a PBS or a 20 mM glucose solution on a bare WE1 or a GOx modified WE1. (**d**) CV responses of PBS or 15 mM lactate solution on a bare WE2 or a LOx-modified WE2. (**b**,**e**) CV responses PBS on WE1 and WE2 at four different scan rates (75, 125, 250, and 500 mV/s). (**c**,**f**) Oxidation current peaks, *I_p_*, plotted against the scan rates from graphs (**b**) and (**e**). (**g**,**h**) The *I_p_* values of 20 mM glucose measured across a wide pH and temperature range. (**j**,**k**) the *I_p_* values of 15 mM lactate measured across a wide pH and temperature range. (**i**,**l**) *I_p_* values of 20 mM glucose and 15 mM lactate measured after various storage times (0, 72, 144, and 216 h). All experiments are conducted on double working screen-printed carbon electrodes (DWSPCEs).

CV responses of PBS or a metabolite (glucose/lactate) on bare or modified DWSPCEs were recorded to evaluate their stabilities. Figure 2a displays the collective CV responses for four conditions (PBS on bare electrodes, PBS on GOX-modified electrode, 20 mM glucose on bare electrode, and 20 mM glucose on GOX-modified electrode) in WE1. Figure 2d displays the collective CV responses for four conditions (PBS on bare electrode, PBS on LOX-modified electrode, 15 mM lactate on bare electrode, and 15 mM lactate on LOX-modified electrode) in WE2. No responsive *I_p_* was recorded in PBS on bare electrode, whereas a responsive *I_p_* was observed in PBS on GOX/LOX-modified electrodes. The highest *I_p_* was generated by 20 mM glucose on GOX-modified electrode for WE1 and 15 mM lactate on LOX-modified electrode for WE2. Here, the CV response was mainly attributed to the following three factors: (i) the deposited enzyme (GOX or LOX), (ii) the mediator (FcMeOH), and (iii) the metabolite (glucose or lactate) concentration in solution, as shown in Figure 1. Evidently, PBS on bare electrode did not produce *I_p_* because all three contributing factors were lacking. In contrast, 20 mM glucose on GOX-modified electrode in WE1 and the 15 mM lactate on LOX-modified electrode in WE2 generated the highest *I_p_* levels because all three contributing factors enhanced the signal.

We further investigated the stabilities of the DWSPCEs using CV across a wide range of scan rates. CV responses of the PBS (10 mM) solution were measured at varying ascending scan rates (75, 125, 250, and 500 mV/s). As shown in Figure 2b,e, *I_p_* for PBS decreased, as the scan rate decreased from 500 mV/s to 75 mV/s. Based on the *I_p_* values obtained from Figure 2b,e, Randles–Sevcik curves for CV were constructed, as shown in Figure 2c,f. Absolute values of *I_p_* were found to follow a linear relationship with the square root of the scan rates, for which correlation coefficients were 0.99 and 0.98 for WE1 and WE2, respectively. A linear relationship implied that the proposed DWE possesses a typical diffusion-controlled electrochemical characteristic. Therefore, these electrodes were demonstrated to be stable for use in further practical quantitative analysis [19].

The stabilities of the electrodes were also evaluated under different temperature and pH conditions (for 24 h). As shown in Figure 2g,k, *I_p_* values for 20 mM glucose and for 15 mM lactate were stably maintained without fluctuation througout the neutral pH range and at temperatures under 45 °C. However, high fluctuations were observed in the *I_p_* value at extreme temperatures higher than 45 °C and at extreme pH levels (highly acidic and basic). The long-term storage ability of DWE was also investigated for up to 216 h at 4 °C. As shown in Figure 2i,l, the *I_p_* value for 15 mM lactate and 20 mM glucose was stably maintained at more than 98% of its initial level throughout the storage time of 216 h. Insignificant variation over long storage times as well as a comparable response over a neutral pH range and at temperatures below 45 °C indicated that the DWE possess excellent stability under normal operating conditions.

### 3.2. Selectivity

**Figure 3:**
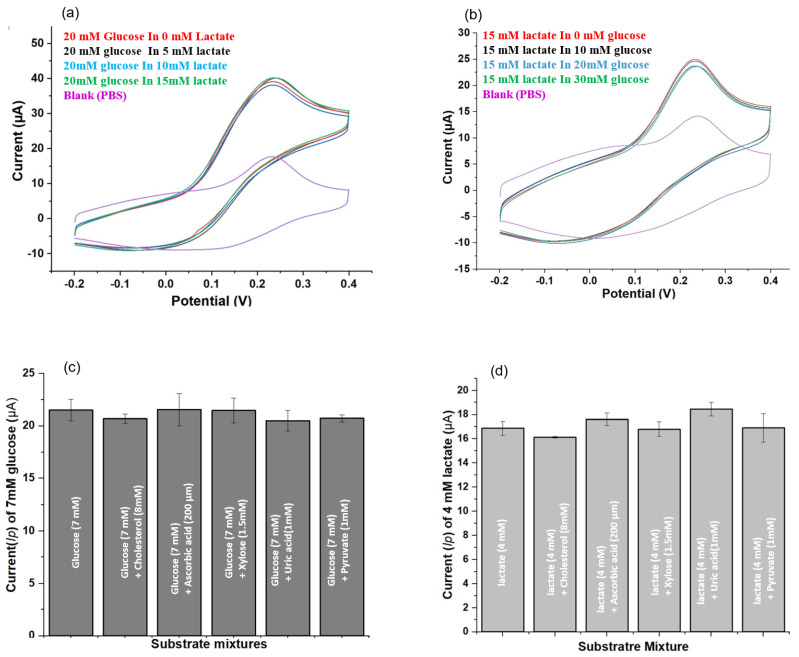
Selectivity test: (**a**) CV responses for 20 mM glucose in different lactate solutions (0 mM, 5 mM, 10 mM, and 15 mM). (**b**) CV responses for 15 mM lactate in different glucose solutions (0 mM, 10 mM, 20 mM, and 30 mM). (**c**,**d**) Bar graph representation of the mean *I_p_* for 7 mM glucose and 4 mM lactate prepared in a separate mixture of 8 mM cholesterol, 200 µM ascorbic acid, 1.5 mM xylose, 1 mM uric acid, and 1 mM pyruvate. All experiments were conducted on a double electrode sensor (GOX and LOX modified DWSPCEs).

The selectivity of the dual electrode biosensor (DEB) was investigated for eight combinations of glucose and lactate. Figure 3a displays the CV responses for 20 mM glucose along with different lactate solutions (0 mM, 5 mM, 10 mM, and 15 mM). Figure 3b displays the CV responses for 15 mM lactate along with different glucose solutions (0 mM, 10 mM, 20 mM, and 30 mM). The calculated mean *I_p_* values for 20 mM glucose and 15 mM lactate concentrations were 20.39 ± 0.09 µA and 7.28 ± 0.06 µA, respectively. Such low levels of standard deviation confirmed that glucose and lactate do not interfere with each other during measurements. The ability of DWE in discriminating a specific biomarker, glucose or lactate, in a mixture of bodily fluids was also investigated. This detection system focuses on determining blood glucose and lactate levels; therefore, normal blood glucose (7 mM) and lactate (4 mM) concentrations were used for comparisons for these experiments. Figure 3c,d summarize the DWE’s ability to selectively measure glucose and lactate from a mixture of separate blood biometabolites. Recovery rates higher than 97% were observed for the DWE, as illustrated by the bars generated from the mean *I_p_* values of glucose (7 mM) and lactate (4 mM) prepared in separate mixture of cholesterol, xylose, uric acid, ascorbic acid, and pyruvate. Therefore, this biosensor exhibits high selectivity for its targeted biomarkers (glucose and lactate) and can be used to measure all three structures (glucose only, lactate only, and glucose and lactate sequentially), all of which are present in a single drop of a blood sample.

### 3.3. Sensitivity

**Figure 4:**
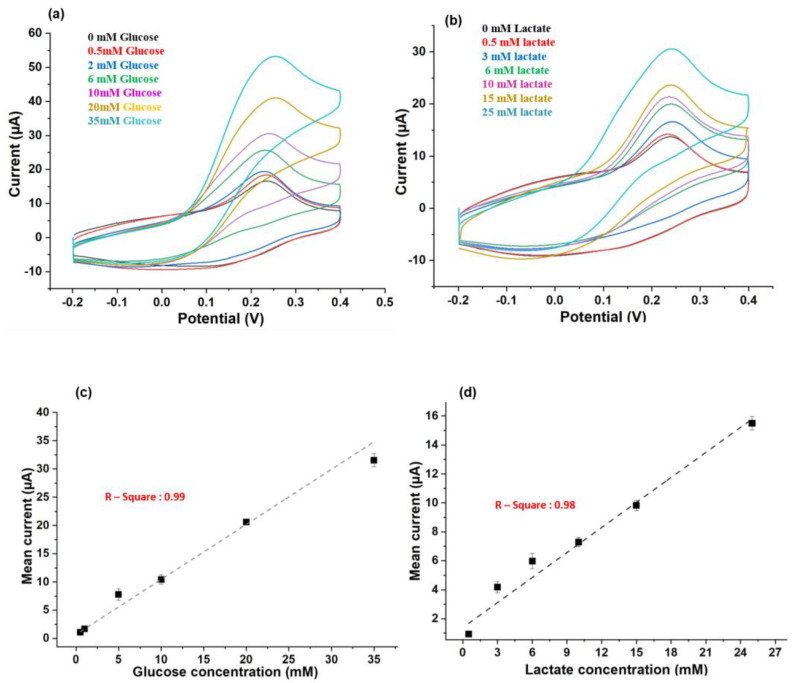
Sensitivity test: (**a**) CV responses of glucose solutions from 0.5 to 35 mM and (**b**) CV responses of lactate solutions from 0.5 to 25 mM. (**c**,**d**) Calibration curves of *I_p_* s with respect to glucose and lactate concentrations, which show a robust linear relationship. The measurement of each condition was conducted in triplicate.

CV responses were recorded across a wide range of concentrations of each metabolite (0.5–35 mM for glucose and 0.5–25 mM for lactate) to evaluate the sensitivity of the biosensor. Figure 4a,b display the CV responses for glucose and lactate from the seven combinations of the metabolites (I–VII). In Figure 4c,d, the calibration curves with error bars displayed a robust linear relationship between glucose and lactate concentrations and their respective *I_p_* values. Here, each *I_p_* value plotted in Figure 4c,d was obtained from triplicated measurements of glucose and lactate. The R-squared values for glucose and lactate were 0.99 and 0.97, respectively. The measurement sensitivities of the DWE for glucose and lactate were 1.22 and 0.65 µA mm^−2^ mM, respectively. This verified that the calibration curves for both glucose and lactate demonstrate good linearity and sensitivity. As shown in Table 1 and Table 2, measurement range and sensitivities of DWE were comparable or superior compared with those of recently developed glucose and lactate meters. These successful measurements across a wide range of concentrations of each metabolite (0.5–35 mM for glucose and 0.5–25 mM for lactate) indicated that the proposed DEB is suitable to quantify both glucose and lactate concentrations, spanning their physiological range in mammals (0.56–35 mM for glucose and 0.5–25 mM for lactate) [20,21].

Supplementary Figure S2a,b display the CV responses for seven combinations of glucose and lactate (I–VII); however, for these readings, the lactate concentrations were measured first, followed by the glucose concentration. The *I_p_* values for all lactate concentrations shown in Supplementary Figure S2b are higher than those shown in Figure 4b, whereas the *I_p_* values for all glucose concentrations shown in Supplementary Figure S2a are lower than those shown in Figure 4a. This decrease in *I_p_* values for glucose could be caused by the desorption-mediated diffusion of iron (Fe) from the electrode surface [22]. Therefore, to avoid such an error, the lactate calibration plot shown in Supplementary Figure S2c should be used when only lactate measurement is required.

**Table 1:** Glucose sensitivity of DWE compared with those of commercial glucose meters and recent reports.

Company/Reference	Range of Measurement (mM)	Sensitivity (µA mm^−2^ mM)
Glucose meter (Freestyle lite)	1.1–33.3	-
Glucose meter (Aga matrix)	1.1–33.3	-
Glucose meter (Nova)	1.1–33.3	-
Glucose meter (Accu-check)	1.1–33.3	-
[23]	0–12	0.16
[24]	0.025–17	0.251
[25]	0.1–5	0.77
[26]	0.1–10	0.75
Proposed DWE	0.5–35	1.22

**Table 2:** Lactate sensitivity of DWE compared with those of commercial lactate meters and recent reports.

Company/Reference	Range of Measurement (mM)	Sensitivity (µA mm^−2^ mM)
EKF diagnostics (Lactate scout 4)	0.5–25	-
Arkray (Lactate pro-2), 2020	0.5–25	-
Arkray (Lactate pro), 2012	0.8–23.3	-
Nova biomedicals (Lactate plus), 2020	0.3–25	-
[27]	1–100	-
[28]	0–1.6	0.16
[29]	0.2–5	-
[30]	0.2–2	0.537
[31]	0.5–10	-
[32]	0.1–5	0.1
[33]	1–1.2	0.08
Proposed DWE	0.5–25	0.65

### 3.4. Efficiency with Whole Blood

#### 3.4.1. Animal Management

**Figure 5:**
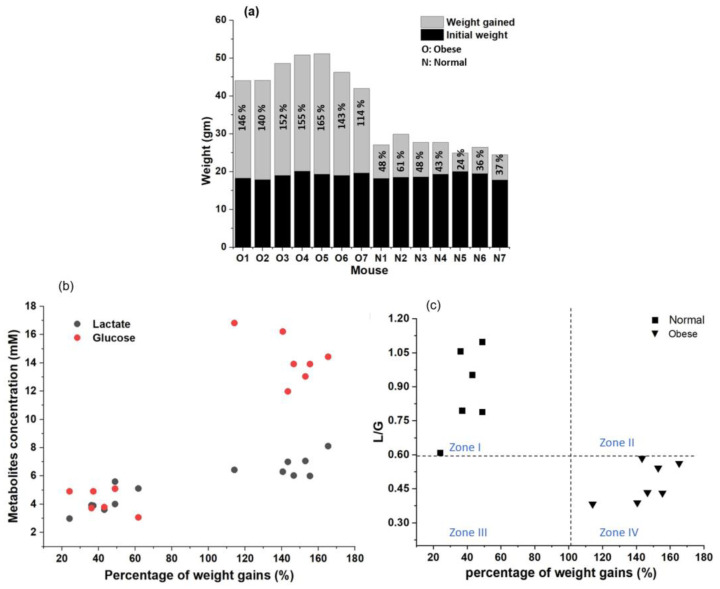
Efficiency test: (**a**) a bar graph displaying body weight gains in obese and normal mice. (**b**) Spotted plot of blood glucose (●) and lactate (●) concentrations with respect to percentages of weight gains. (**c**) Spotted plots of lactate to glucose ratio (L/G) calculated from plot (**b**) with respect to the percentage of weight gain. Here, the symbols ■ and ▼ represent normal and obese mice, respectively. All measurements (percentage of weight gains, and blood glucose and lactate) were performed after 28 weeks of diet (normal or HFD). The L/G plot is divided into four subsections: zone I, zone II, zone III, and zone IV in (**c**).

We prepared seven obese and seven normal mice by feeding them with high fat diet (HFD) and normal diet (ND). Figure 5a presents the percentages of weight gains for all fourteen mice after 28 weeks of feeding. Initial weights for both groups were similar, which were 19.03 gm for the obese group and 18.85 gm for the normal group. The average weights gained for normal and obese groups were 8.04 ± 1.95 gm (percentage of weight gain: 24–61%) and 27.68 ± 3.04 gm (percentage of weight gain: 114–165%). HFD contributed to a higher weight gain than ND. Previous work conducted by our group have shown that normal mice can reach the status of obesity within 12 weeks of feeding with this HFD [18]. As 12 weeks may not be enough to provide effective changes in blood lactate, we continued HFD and ND feeding for each group up to 28 weeks to see the changes in lactate [34].

#### 3.4.2. Blood Glucose and Lactate

Venous blood was extracted from seven obese and seven normal mice after 28 weeks of feeding with HFD and ND, respectively. In Figure 5b, spotted plots represent glucose and lactate concentrations in whole blood samples with respect to percentage of weight gain for each mouse. The glucose and lactate concentrations from the obese group were 14.33 ± 1.57 mM and 6.7 ± 0.69 mM, respectively, while the glucose and lactate concentrations from the normal group were 4.37 ± 0.76 mM and 4.16 ± 0.82 mM, respectively. Distinguishable clusters were formed for glucose (red) and lactate (black), which could differentiate the obese group from the normal group. Table 3 and Table 4 compared the glucose and lactate concentrations obtained from our DWE biosensor with previously reported glucose and lactate concentrations in blood. These values of glucose and lactate concentrations from normal mice and obese mice in our results were comparable with those of previously reported concentrations [33,34,35,36,37,38,39,40,41,42,43,44,45]. Increasing ratios were calculated with the following equation: $\;\mathrm{Increasing}\;\mathrm{ratio}=\frac{Obese-Normal}{Normal}\times 100$. As shown in Table 3 and Table 4, increasing ratios of glucose and lactate became higher with an increase in the HFD period. To the best of our knowledge, we obtained the highest increasing ratios for both glucose and lactate because we fed these mice for up to 28 weeks, which would be the longest one reported so far. Glucose increase is a well-known phenomenon in HFD-induced obesity [35]. However, changes in lactate were not significant during obesity monitoring because a relative short term of diet such as 8~12 weeks usually provided a negligible increase (0.5 mM, equivalent to 10% of increasing ratio) in lactate in the case of HFD-induced obesity [33,36,41]. Thus, our high increase (2.5 mM, equivalent to 61% of increasing ratio) of lactate in HFD-induced obesity would be a turning point to consider lactate as an important factor for obesity monitoring [42].

We suggested a new monitoring parameter in blood, the ratio of lactate to glucose (L/G), which reflects the influences of both glucose and lactate on obesity as a single parameter. Similar approaches have been reported in embryo studies, where concentrations of both lactate and glucose are used in single parameter to assign the quality of embryo according to this parameter [9]. To the best of our knowledge, no researcher has previously reported the use of L/G in blood for monitoring health or disease conditions including obesity. Here, by using L/G obtained from Figure 5b, we constructed a “diagnosis plot” for obesity, which categorized mouse conditions onto four different zones (zone I, zone II, zone III, and zone IV) as shown in Figure 5c. The ranges of L/G for normal and prolonged obese mice groups were 0.6–1.09 and 0.38–0.58, respectively. The L/G value of 0.59 could be a clear boarder line between normal and obese mice because the lowest L/G for normal mice was 0.6 and all obese mice had L/G values below 0.59. Similarly, the ranges of the percentage of weight gain for the normal and prolonged obese mice groups were 24–61% and 114–165%, respectively. Thus, the border line percentage of weight gain for distinguishing normal and obese could be between 61% and 114%. Here, we chose 102%, the mid-point of ranges, as the border line.

Zone I (normal cases) was located at the region with a higher L/G ratio (higher than 0.59) and a lower body weight gain (lower than 102%). It was categorized as a “safe zone” in terms of obesity. The higher value of L/G could be explained by the relatively less change of glucose in normal cases compared with that in obese cases (Zone IV) in which glucose, the denominator, dramatically changed. Zone IV (obese cases) was located at the region with a lower L/G ratio (lower than 0.59) and a higher body weight gain (higher than 102%; obese cases). It was categorized as a “danger zone” in terms of obesity. The lower value of L/G, as we mentioned in zone I, could be explained by the relative larger increase in glucose than the increase in lactate because of obesity. Zone II was located at the region with a higher L/G ratio (higher than 0.59) and a higher body weight gain (higher than 102%). Despite obesity, this zone could be safe. However, it was on the way to the danger zone (zone IV) because L/G ratio was still higher than the border line of 0.59. Zone II could be considered as “transition zone”. If cases keep having higher weight gains, blood glucose levels will gradually increase because of a higher pancreatic insulin release, leading to insulin resistance [46], which eventually causes a decrease in the L/G ratio (lower than 0.59) and the conversion to a danger zone (zone IV). Zone III was located at the region with a lower L/G ratio (lower than 0.59) and a lower body weight gain (lower than 102%). Despite their normal weight conditions, zone III could be considered “unhealthy” because the L/G ratio was lower than the border line of 0.59. Zone III is rarely possible in obese studies. It is categorized as a “rare zone”. If the normal group has additional clinical conditions such as hereditary diabetes, such cases could be considered zone III because hereditary diabetes relatively increases blood glucose independent of body weight, ultimately decreasing the L/G ratio [47].

## 4. Conclusions

Here, we reported a DWE biosensor for the sequential measurement of glucose and lactate concentrations in a single drop of whole blood from normal mice and prolonged obese mice. The DWE biosensor consists of a single reference electrode and a counter electrode with two carbon working electrodes: WE1 (modified with GOx) and WE2 (modified with LOx). The quantification of glucose and lactate was accomplished with the measurement of enzyme-catalyzed oxidation of metabolites with the cyclic voltammetry (CV) method. We evaluated this DWE biosensor by quantifying glucose and lactate levels in respective standard solutions and whole blood extracted from mice. The biosensor showed a high stability, selectivity, sensitivity, and effectivity. The values of glucose and lactate concentrations obtained with the DWE biosensor could differentiate the obese mice group from the normal mice group. These values were comparable with those of previously reported concentrations [33,34,35,36,37,38,39,40]. Using glucose and lactate concentrations in normal and obese cases measured with the biosensor, L/G was calculated and used to obtain a new obesity diagnostic plot. The proposed diagnostic plot used L/G ratios and weight gain percentages to differentiate the diagnostic plot onto four different zones. The diagnostic plot can be used to monitor obesity conditions with L/G. It could be used for determining clinical prognosis. Additional studies for changes in glucose and lactate caused by diet and other factors such as habits, addictions, diseases, and so on [48,49] can provide a critical point for prediction and warn about the onset and progression of a disease by enhancing the “diagnostic plot”. Thus, we believe that our biosensor and diagnostic plot are promising tools for monitoring general health conditions as well as obesity using glucose and lactate together.

## Figures and Tables

**Table 3:** Comparison of glucose concentrations in blood samples of mice with previously reported concentrations.

HFD Period (Weeks)	Sampling	Glucose Concentrations (mM)			References
		Normal Mice	Obese Mice	Increasing Ratios	
12	Plasma	6.1	10	64%	[34]
8	plasma	7.6	8.8	15%	[35]
12	Whole blood	5.7 ± 0.2	9.6 ± 0.3	70%	[36]
13	Plasma	5.9	10	69%	[37]
24	Serum	6.7	16.7	150%	[38]
28	Whole blood	4.3 ± 0.7	14.3 ± 1.5	228%	Our result

**Table 4:** Comparison of lactate concentrations in blood samples of mice with previously reported concentrations.

HFD Period (Weeks)	Sampling	Lactate Concentrations (mM)			References
		Normal Mice	Obese Mice	Increasing Ratios	
NA	Whole blood	4.5	NA	NA	[39]
NA	NA	4.6 ± 0.7	NA	NA	[40]
8	plasma	4.8	5.58	10%	[33]
13	plasma	4.5	5.01	11%	[36]
28	Whole blood	4.16 ± 0.82	6.7 ± 0.69	61%	Our result

## Data Availability

Not applicable.

## Supplementary Materials

The following are available online at https://www.mdpi.com/article/10.3390/bios11120507/s1, Figure S1: Methodology optimization: (a) CV responses of glucose solutions from 200 mg/dL to 600 mg/dL on GOX-deposited SPCES and (b) CV responses of lactate solutions from 0.5 to 10 mM on LOX-deposited SPCES. (c) Difference in *I_p_* generated by 35 mM and 20 mM glucose on DWE prepared with linear Gox concentrations (100 U/mL to 600 U/mL). (d) Difference in *I_p_* generated by 25 mM and 15 mM lactate on DWE prepared with linear LOX concentrations (100 U/mL to 400 U/mL). The measurement of each condition was conducted in triplicate, Figure S2: Desorption study: (a) CV responses of glucose solutions from 0 to 35 mM on DWE, measured after generation of CV response for lactate present on the same solution. (b) CV responses of lactate solutions from 0.5 to 15 mM on DWE, measured before glucose solution present on the same solution (c) Calibration curve for lactate generated using the *I_p_* of (b). The fluctuation in *I_p_* of glucose and lactate was potentially caused by desorption-mediated diffusion.
