# Supplementary material for: A Dual Electrode Biosensor for Glucose and Lactate Measurement in Normal and Prolonged Obese Mice Using Single Drop of Whole Blood
Source: Biosensors (Basel). 2021 Dec 9;11(12):507. doi: 10.3390/bios11120507 (PMC8699454; doi:10.3390/bios11120507)
Supplement: Supplementary file 1 [file biosensors-11-00507-s001.zip › biosensors-1479975-supplementary.pdf]

biosensors

MDPI

Article

A Dual Electrode Biosensor for Glucose and Lactate Measurement in Normal and Prolonged Obese Mice Using Single Drop of Whole Blood

Mukesh Thapa, Ryong Sung and Yun Seok Heo *

Department of Biomedical Engineering, School of Medicine, Keimyung University, 1095 Dalgubeol-daero, Dalseo-gu, Daegu 42601, Korea; 1004672@stu.kmu.ac.kr (M.T.); rs.ryongs@gmail.com (R.S.)
* Correspondence: yunsheo@kmu.ac.kr

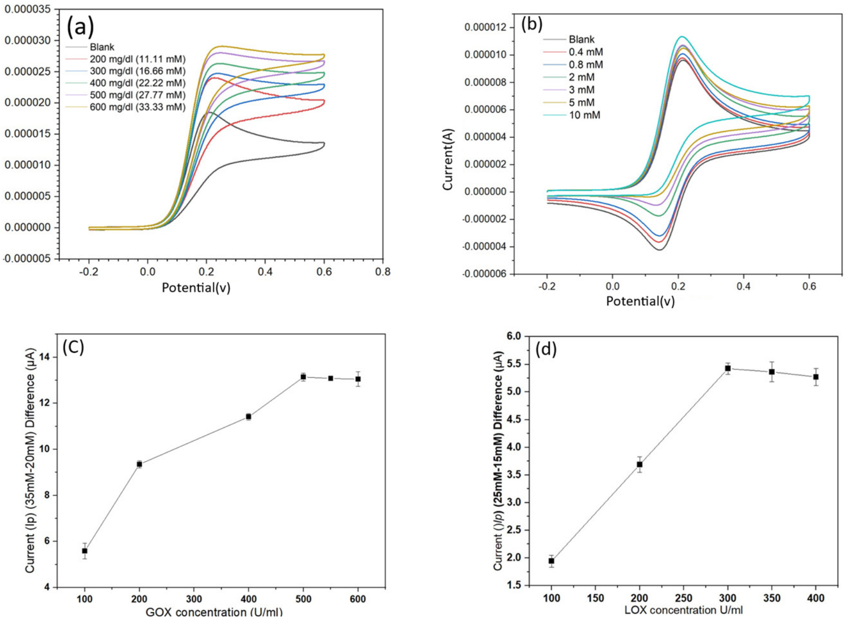

Figure S1. Methodology optimization: (**a**) CV responses of glucose solutions from 200mg/dl to 600mg/dl on GOX deposited SPCES and (**b**) CV responses of lactate solutions from 0.5 to 10 mM on LOX deposited SPCES. (**c**) difference of Ip generated by 35 mM and 20 mM glucose on DWE prepared with linear Gox concentration (100 U/mL to 600 U/mL). (**d**) difference of Ip generated by 25 mM and 15 mM lactate on DWE prepared with linear LOX concentration (100 U/mL to 400 U/mL). The measurement of each condition was conducted in triplicate.

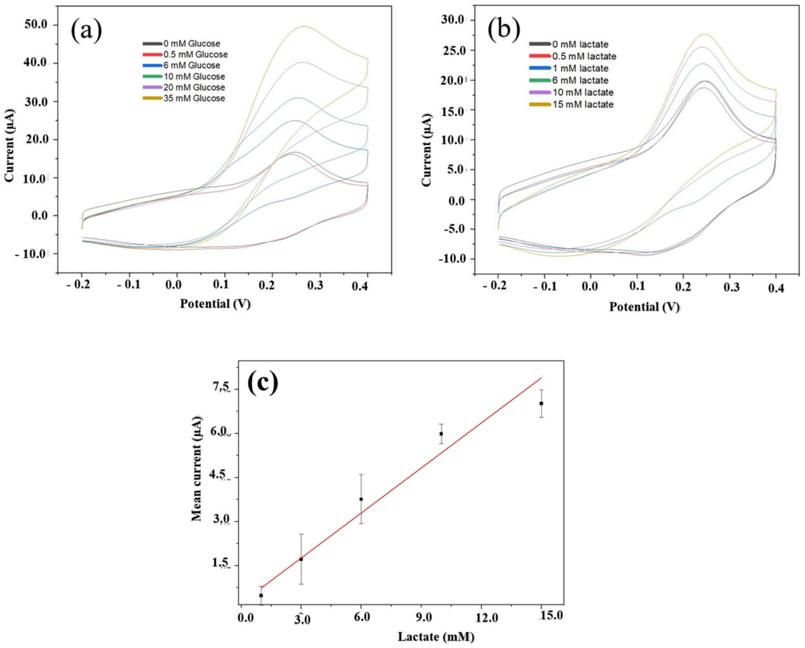

Figure S2. Desorption study: (**a**) CV responses of glucose solutions from 0 to 35 mM on DWE, measured after generation of CV response for lactate present on same solution. (**b**) CV responses of lactate solutions from 0.5 to 15 mM on DWE, measured before glucose solution present on same solution (**c**) calibration curve for lactate generated using Ip of (b). The fluctuation in I_p of glucose and lactate was potentially caused by desorption -mediated diffusion.
